# Effects of ambient noise on zebra finch vigilance and foraging efficiency

**DOI:** 10.1371/journal.pone.0209471

**Published:** 2018-12-31

**Authors:** Julian C. Evans, Sasha R. X. Dall, Caitlin R. Kight

**Affiliations:** 1 Centre for Ecology and Conservation, University of Exeter, Cornwall Campus, Penryn, United Kingdom; 2 University of Exeter, Exeter, United Kingdom; University of Salford, UNITED KINGDOM

## Abstract

Ambient noise can affect the availability of acoustic information to animals, altering both foraging and vigilance behaviour. Using captive zebra finches *Taeniopygia guttata*, we examined the effect of ambient broadband noise on foraging decisions. Birds were given a choice between foraging in a quiet area where conspecific calls could be heard or a noisy area where these calls would be masked. Birds foraging in noisy areas spent a significantly more time vigilant than those in quiet areas, resulting in less efficient foraging. Despite this there was no significant difference in the amount of time spent in the two noise regimes. However there did appear a preference for initially choosing quiet patches during individuals’ second trial. These results emphasise how masking noise can influence the foraging and anti-predation behaviour of animals, which is particularly relevant as anthropogenic noise becomes increasingly prevalent in the natural world.

## Introduction

Humans have dramatically altered the temporal, spectral, and spatial aspects of the world’s soundscape [1–6]. These anthropogenic noises are generally characterised by higher amplitudes and lower spectral frequencies than those typically found in nature, which reduces the ability of many animals to distinguish signals and cues from background noise [7, 8]. Many animals utilise acoustic cues and signals as sources of information while carrying out a variety of behaviours. These can include the vocalisations of conspecifics whilst searching for a mate [9] or the sound of approaching predators when attempting to avoid predation [10]. Missing these cues and signals could result in missed mating opportunities, starvation, injury, or even death. As such, the disruption of an animal’s ability to receive cues and signals by anthropogenic noise might have significant impacts on an animal’s behaviour.

A number of studies have investigated how animals change their signalling strategies in response to anthropogenic noise by adjusting their signal’s amplitude or pitch [11–16]. Another important aspect to consider is the effect of noise on the potential receivers of acoustic signals, or the impacts of loud noise on vital non-communication behaviours such as foraging [6, 17–22]. If acoustic signals and cues are unavailable, animals may be able to use alternative sources of information, but this can impact other behaviours. For example, many animals will use alarm signals or the sound of approaching predators as a source of information about predation risk, allowing less time to be spent scanning for predators and more time for other activities such as foraging [23–25]. The masking of these signals and cues by high levels of background noise could therefore increase predation risk, forcing animals to compensate by spending more time scanning for visual cues of predation risk [21, 22, 26]. This increase in vigilance will reduce time available for other behaviours [18, 26, 27]. Additionally, animals that are “distracted” by the presence of background noise may make sub-optimal decisions about predator avoidance or foraging [21, 28–33]. In both cases, the presence of high levels of background noise could have fitness consequences due to missed opportunities, injury or predation [21, 26, 32].

Studies of noise and predation avoidance have reported significant increases in time spent vigilant and other changes in anti-predator behaviour [18, 21, 24, 26]. There is also evidence that noisy conditions lead to decreases in food intake [18, 24, 28]. Optimal foraging theory states that an individual will attempt to maximise its net energy gain by leaving a patch once intake drops below a critical threshold [34, 35]. If noise reduces foraging intake rates then we would expect animals to spend more time in quiet areas than noisy ones. Animals have been shown to avoid noisy areas when making breeding habitat decisions [36, 37], but few studies exist examining how noise affects choice of foraging location (but see: [6, 20, 38–40]).

We examine this using a captive population of zebra finches *Taeniopygia guttata*. Zebra finches are social and highly gregarious; while foraging in a group, they constantly communicate with flock-mates using contact calls that could potentially be masked by background noise. Contact calls help keep a group cohesive and allow individuals to keep track of the locations of other group members even when they cannot be seen [41]. As individual zebra finches have been shown to respond negatively to perceived isolation [42], and because of the negative behavioural trade-offs associated with noise, we expected that individuals would spend less time foraging when in noisy areas and that individuals would choose to forage in quieter areas when given the choice.

## Methods

Methodological and animal welfare issues were approved by the Ethical Committee of the University of Exeter and discussed with our Home Office inspector, who agreed that no special licence was required. The condition and health of all birds were monitored on a daily basis. Except when participating in trials, the birds were housed in an outdoor aviary in two single-sex cages containing an average of ten birds apiece. Food and water were provided *ad libitum* and the birds had access to nest boxes in which they could roost during cool weather. Outdoor temperatures ranged from 4.0°C to 26.5°C. Only females were used in experiments as these individuals were less likely than males to engage in territorial behaviours such as singing or displaying during experimental trials (Dall, personal observation). A total of 20 individuals were tested, with ages of either 3 (n = 13) or 4 (n = 7) years old. While engaged in testing, birds were moved six at a time to an indoor aviary which had a light:dark cycle of 14:10 hours and an average temperature of 19.5°C. Birds were always moved the day before trials in order to allow them to acclimatise to the different environmental conditions.

**Fig 1:**
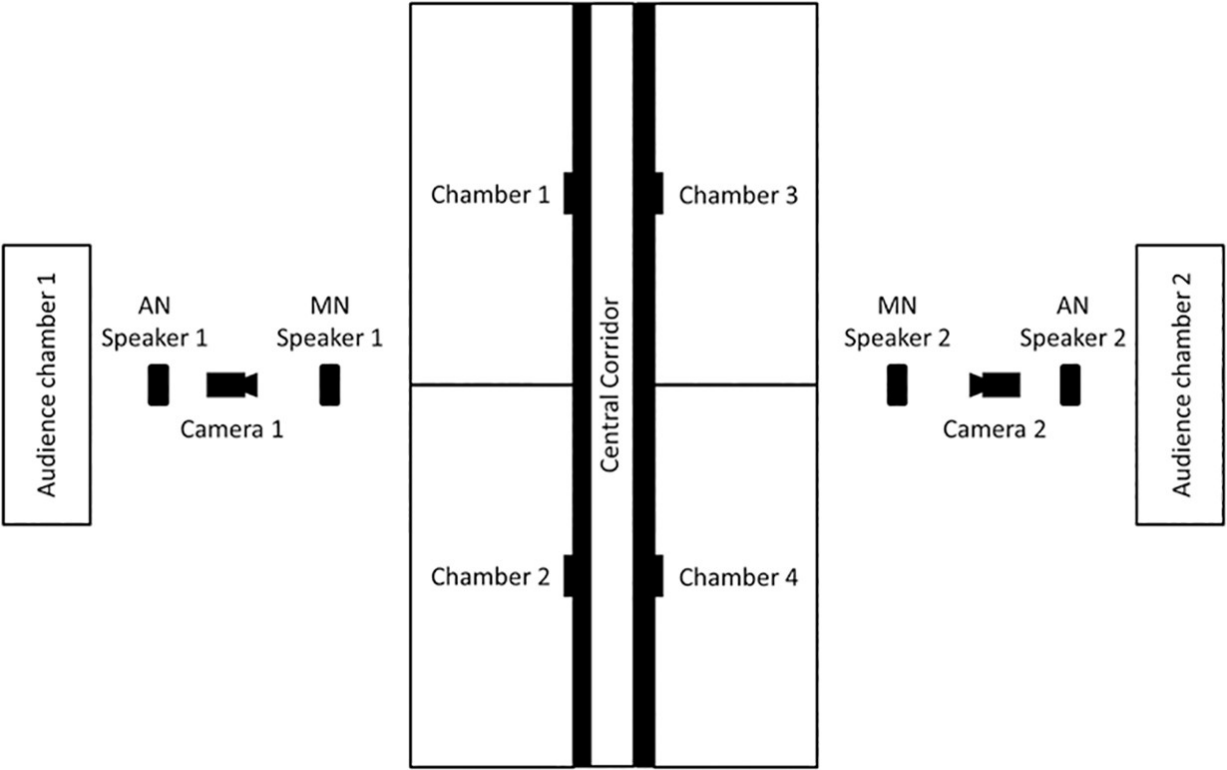
Layout of test arena. Diagram (not to scale) showing positioning of cameras, audience noise (AN) speakers and masking noise (MN) speakers. Thicker walls surrounding the central corridor represent those supplemented with soundproofed foam.

To better replicate the auditory conditions of flocks in the wild [42], birds in the indoor aviary were placed in cages 1.2 m away and 1.24 m above the test arena (Fig 1). From this location (which was not visible to a bird in the test chamber), these birds could deliver contact calls to an individual in the test arena. The contact calls of the live audience were supplemented with a recording of conspecific zebra finch calls recorded in the outdoor aviary. These calls were played continuously from two MP3 players attached to portable speakers (Sandisk Sansa clip+ Mp3 player, Milpitas, USA; Portable Sound Laboratories iMaingo 2 speakers, Agoura Hills, USA) positioned 0.5 m from the arena (Fig 1).

The test arena consisted of a central corridor (0.3 × 0.3 × 1.7 m) with two chambers on either side (0.7 × 0.8 × 0.3 m). Each of these chambers was accessible from the central corridor by a single door (0.18 × 0.2 m). This design was intended to make the foraging environment sufficiently complicated so that a bird’s choice was not simply based on a preference for the right or left side of the arena. The ceilings of all four chambers were composed of a thin wire mesh that prevented birds from escaping but allowed a clear line of sight for a camera (Sony Handycam DCR-SR37, Tokyo, Japan) on each side of the arena (Fig 1).

Each chamber contained water, grit, sloping walls (to prevent birds from moving out of sight of cameras) and a food tray containing seed covered with approximately 0.5 cm of aviary bird sand. The amount of food provided per tray was equivalent to half a bird’s average daily intake. Grit and water were also available in the central corridor.

Acoustic foam was installed in both the foraging chambers and central corridor to maintain a difference of 20 dB (sound pressure level; SPL) between the two sides of the arena.

Experimental ambient noise, consisting of an artificial broadband noise centred around a 450-Hz tone (ranging from 200-Hz to 800-Hz) was generated in Audacity ([43], S1 Fig.) and broadcast from another two MP3 players attached to portable speakers placed at a distance of 0.25 m from the exterior wall of the test arena (Fig 1). The tone overlapped with the mean frequency of female zebra finch contact calls (400 Hz to 500 Hz, Zann [42], Vignal, Mathevon [44]), meaning that our ambient noise at least partially masked the vocalisations of zebra finches. During the first and last 20 seconds of each ambient noise recording, the volume was slowly faded in and out, respectively, to avoid startling the birds. Noise levels were compared using a digital sound level meter (Dick Smith Electronics Digital Sound Level Meter Q 1362, Sydney, Australia), calibrated to report “real-world” values using tones of known amplitude and frequency [45, 46]. The maximum amplitude of the “noisy” noise regime was 70 dB (SPL, A), comparable to levels achieved by heavy traffic or vegetation movement caused by high wind speeds [47]. In comparison, noise levels in the “quiet” treatment on the other side of the arena were 50 dB (SPL, A) while the noise was playing.

Prior to the beginning of testing, all birds were given two hours to explore the test arena in groups. At the beginning of each trial (after the difference in noise on either side of the arena had been checked), a focal bird was chosen at random and was placed in the central corridor of the testing arena for two hours. During this time, the doors to all four adjoining chambers were closed. This time allowed the focal individual to recover from the stress of being handled and relocated, and deprived the bird of food so as to encourage foraging once the trial began. At the end of the two hours, the doors to the side chambers were opened and playback of ambient noise began on one side of the arena (randomly selected by coin toss). After 30 minutes, the noise regime was reversed so that the initially exposed side remained quiet (50 dB (SPL, A)) and the other side was exposed to noise. Each trial lasted a total of one hour. Every bird was tested twice, with at least one day of rest before the second trial. During the second trial, the order in which sides of the arena were exposed to noise was the opposite of the previous trial.

When evaluating footage of the trial, we focused on three behaviours. First, we examined whether birds showed an initial preference for a quiet foraging chamber when first entering one of the side chambers from the central corridor. Second, we explored whether focal individuals cumulatively spent more time in quiet or noisy chambers over the course of the trial. Third, we analysed the amount of time each birds spent vigilant, foraging, in flight or engaged in other behaviours such as preening. Vigilance was defined as holding the head up and looking at the surrounding environment and foraging was defined as looking down toward the foraging tray. As with other studies examining vigilance during foraging, we assumed that birds were unable to gather visual information about the surrounding environment while in the head-down posture [18, 48].

We tested how noise affected foraging choices and behaviour by fitting generalised linear models with binomial error structures in R using the package lme4 [49, 50]. The first model fitted initial choice of noise regime as a binary response variable, in relation to a bird’s age and trial number. Three other models examined the proportion of time a bird spent; in a noise regime, vigilant, and foraging, in response to type of noise regime, trial number, and age. All proportions of time were measured as the proportion of total time spent in areas of the test arena other than the central corridor. All combinations of predictors and their two-way interactions were tested, with individual ID included as a random effect. Response variables were all rescaled and grand mean centred before being modelled. All models were fitted using binomial error structures and AICc scores used to decide which combination of fixed effects best predicted within-site correlation (Bartoń 2016). Where there were multiple candidate models within Δ2AICc of the top model, model averaging was carried out [51]. Additionally, we tested whether birds exhibited a preference for initially choosing quiet patches in a particular trial, using a permutation test which compared the number birds that chose the quiet side of the test arena in a trial in relation to what would be expected if the choice was random. To do this, we generated 1000 random choices for each individual that left the centre corridor in each trial.

## Results

**Fig 2:**
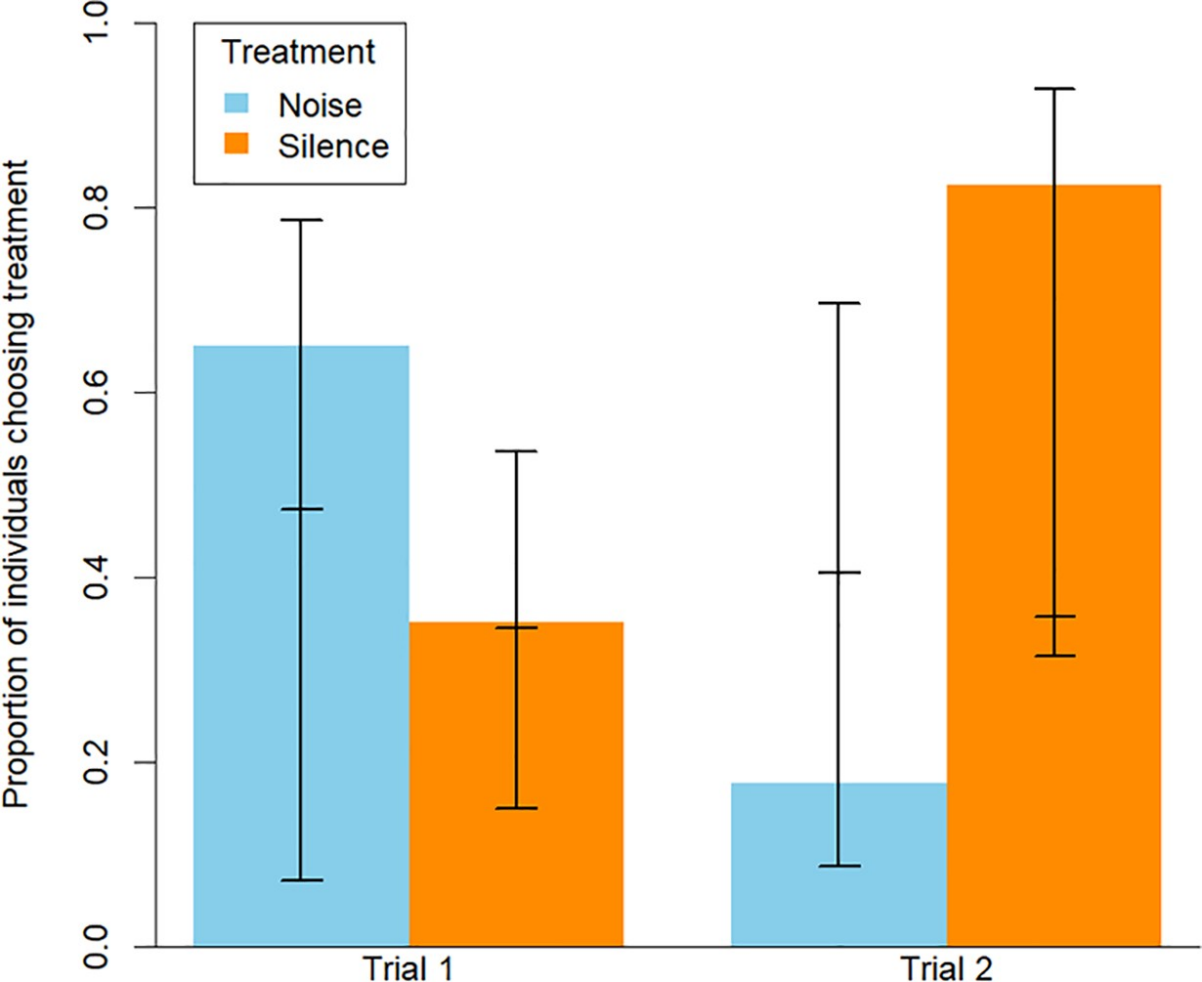
Initial chamber choice. Graph shows proportion of individuals initially choosing a quiet chamber of a noisy chamber in each trial, with standard error.

**Fig 3:**
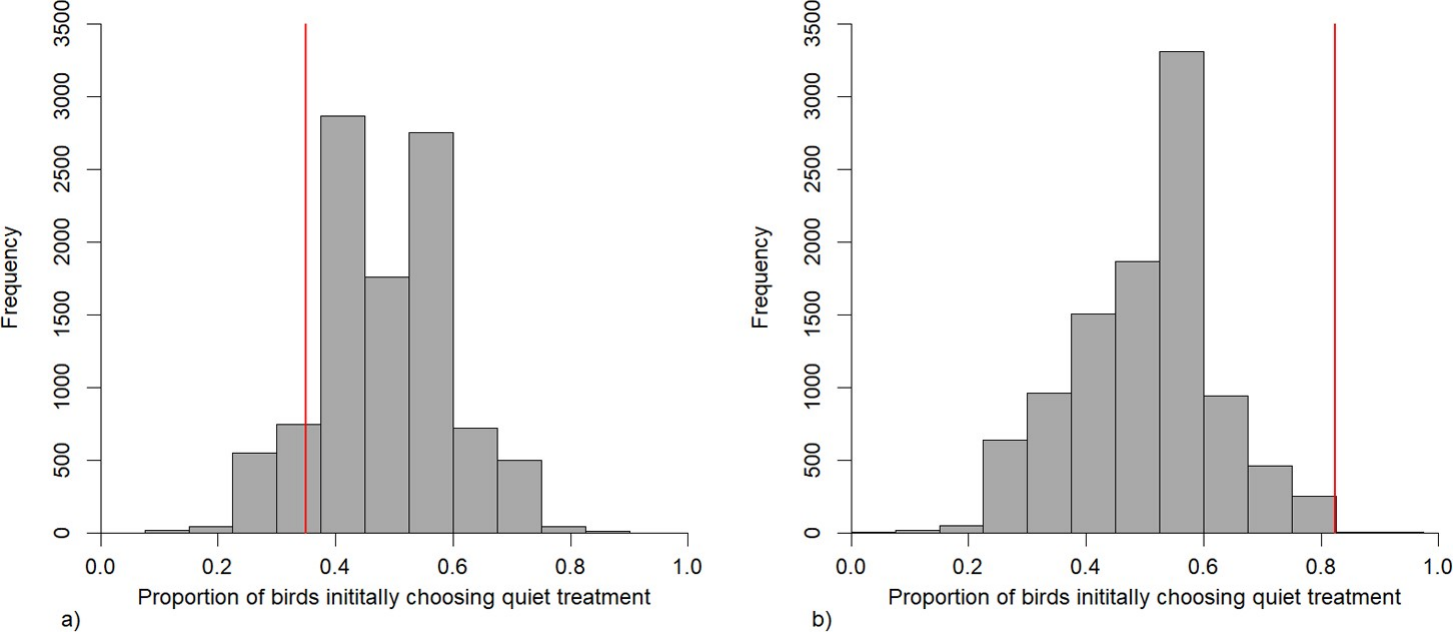
Results of permutation tests. Results of permutation tests comparing the actual proportion of birds initially choosing a quiet patch (red line) to that of 1000 random choices in a): trial 1, b) trial 2.

All 20 birds carried out the first trial, but in the second trial 3 birds remained in the central corridor and were therefore not considered in the analysis. Models of choice of initial noise regime suggested that birds were significantly more likely to choose to first enter a quiet patch in the second trial (Table 1, Fig 2). Similarly, comparing our results to those generated by 1000 random permutations indicated that while birds displayed no preference during the first trial, in the second trial significantly more birds initially chose the quiet noise regime than would have been expected if the choice was purely random (Fig 3). However time spent in a chamber was found to not to be related to ambient noise level or any of the other explanatory variables included in the full model, with the most parsimonious model being the null model (S1 Table).

**Table 1:** Model averaged estimates of models within 2 ΔAICc of the top model (See S2 Table) predicting the probability of birds initially choosing a chamber within the quiet noise regime, with 95% confidence intervals, based on the models within.

Parameter	Importance	Estimate	2.5% Confidence Interval	97.5% Confidence Interval
Intercept		-0.45	-1.60	0.70
Age (4)	1	-0.77	-3.32	0.41
Trial (2)	2	2.39	0.46	4.32

**Fig 4:**
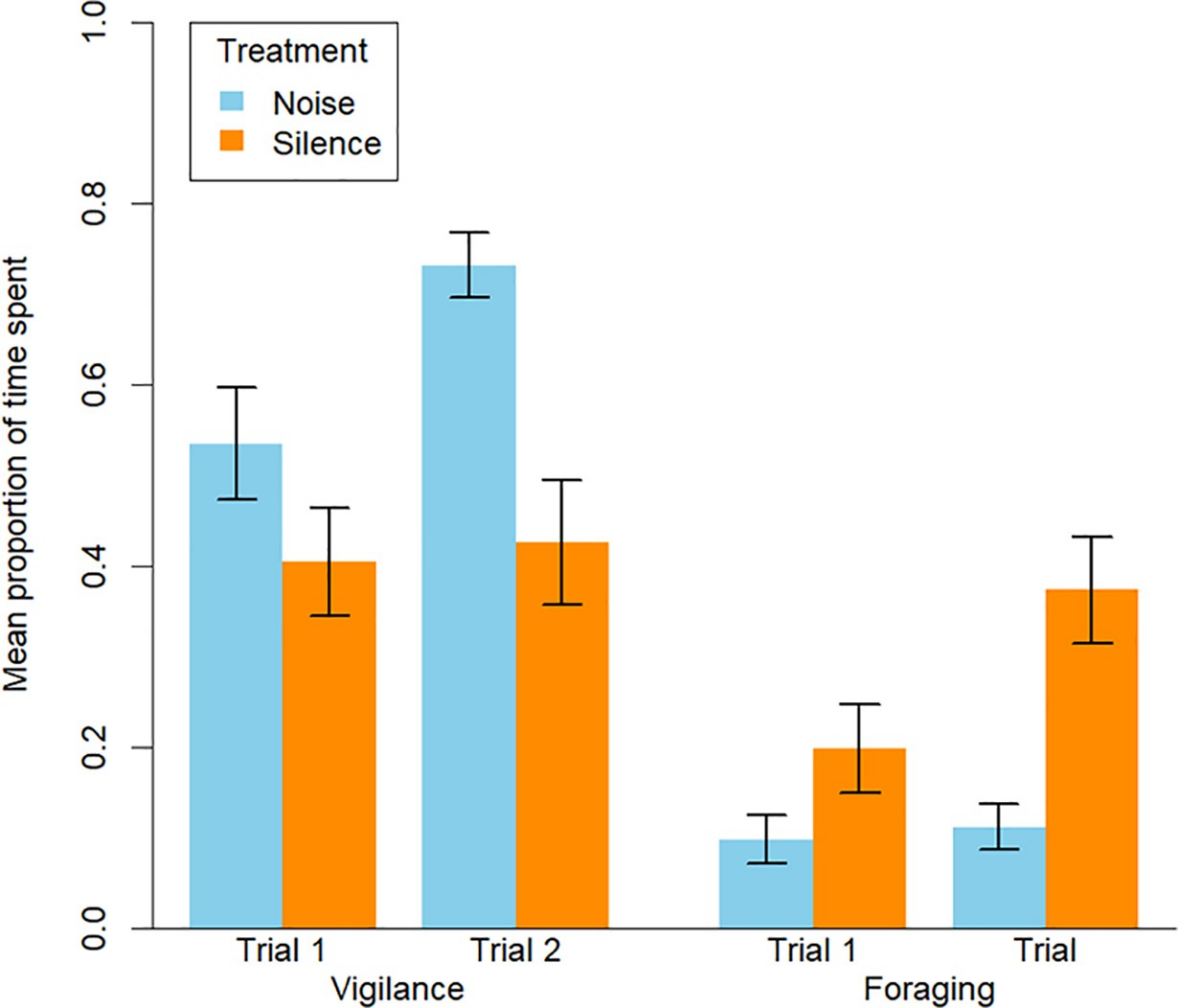
Changes in foraging and vigilance behaviour. Graph showing the effect of noise regime and trial number on the mean proportion of time spent vigilant and foraging, with standard error.

Time budgets appeared to be impacted by ambient noise levels, with significantly more time spent vigilant in noisy patches (Table 2, Fig 4), resulting in significantly less time spent foraging (Table 3, Fig 4). Time spent foraging varied between trials, with birds spending slightly more time foraging in their second trial. However, trial number did not affect the proportion of time spent vigilant (Fig 4).

**Table 2:** Model estimates of top model predicting the proportion of time spent vigilant, with 95% confidence intervals.

Parameter	Estimate	2.5% Confidence Interval	97.5% Confidence Interval
Intercept	0.62	0.54	0.71
Snd (Silence)	-0.22	-0.33	-0.10

**Table 3:** Model averaged estimates of top models predicting the proportion of time spent foraging, with 95% confidence intervals, based on the models within 2 ΔAICc of the top model (See S3 Table).

Parameter	Importance	Estimate	2.5% Confidence Interval	97.5% Confidence Interval
Intercept		0.09	0.01	0.17
Snd (Silence)	2	0.17	0.08	0.26
Trial (2)	1	0.03	0.02	0.18

## Discussion

Our study supports the hypothesis that ambient noise results in altered foraging behaviours. There are several potential explanations for these results. Impacted individuals may perceive the environment as riskier when they are no longer able to hear conspecifics. Individuals may also be unable to make efficient foraging or anti-predation choices or suffer increased stress while in a noisy environment. Alterations in feeding patch choice and behavioural time budgets associated with feeding can reduce foraging efficiency and impact predator detection, though this was not directly tested in this experiment. Similar changes in anti-predator behaviour have been reported in a range of different species [18, 24, 29, 32, 33] suggesting that these changes in behaviour are relevant across a range of taxa.

Almost all birds chose a foraging patch and remained there until the end of the trial, despite changes in background noise. The birds may have been unwilling to relocate because exploration might have been deemed riskier or less efficient than remaining within a known patch, even if a change in ambient noise regime suddenly rendered it less “safe” [52]. Having previously experienced quieter conditions in a particular patch, a bird might also assume that the patch would eventually become quiet again, regardless of current noise levels. This might be particularly true since the finches had previously heard the contact calls of conspecifics in or near that patch. Despite this, a higher number of birds actively chose to initially enter a quiet patch during the second trial. After their experiences during the first trial, birds may have associated a noisy patch with reduced foraging intake or lower levels of safety, and chosen to avoid it [53]. Zebra finches might also be less sensitive to the type of artificial noise used in this study and therefore less likely to move to a different patch [54]. Alternatively, increased sensitivity might result in a freeze response also making it unlikely to switch patches whilst under the noise treatment.

Zebra finches showed an increase in vigilance behaviour in noisy conditions. Time spent vigilant did not decrease during the second trial, suggesting that birds did not become habituated to the noise. We therefore suggest that the increase in vigilance in noisy areas was not a neophobic response. The change in behaviour observed may be due to an inability to hear the contact calls of conspecifics or detect auditory cues of predators approaching. Like many other species, zebra finches likely utilise both visual and auditory social information from conspecifics when assessing predation risk [55]. A head-down foraging posture results a bird’s visual information about its surroundings being reduced or entirely unavailable [48, 56, 57]. In this case, zebra finches should rely heavily on auditory signals and cues, both from conspecifics and approaching predators [18, 48, 58]. In the absence of social information from conspecifics, individuals will only have access to their own personal information [55]. This results in more time spent scanning for predators, which can lead to reductions in foraging efficiency [18, 21, 24, 27].

Alternatively, individuals might be “distracted” by the presence of masking noise, diverting a limited amount of attention to the noise itself. This could cause them to make sub-optimal foraging decisions which increases the amount of time required to forage due to taking longer to find and handle food items [30, 59]. A distracted individual might also require longer to scan for predators as the distraction causes them to take longer to process visual information [29, 33, 60]. In our experiment, mean time spent foraging was significantly lower under the noisy regime, while vigilance increased. This could support the idea that scanning requires more time under the distracting effect of noise, rather than foraging requiring more time. We did not test birds’ foraging intake so are unable to assess the number of errors made in the different noise regimes [31, 33]. In future experiments recording body condition before and after trials would help better quantify the impact of noisy conditions on foraging efficiency. Another potential explanation for changes in behaviour is that individuals might be undergoing increased stress or annoyance due to the noise, causing a reduced motivation to move or forage [59]. However, previous studies suggest that our noise regime of 70 dB might be insufficient to cause a stress response [18]. Additionally, if changes in behaviour are caused by stress or annoyance we might expect some degree of habituation over the course of the trials [18, 61], though potentially birds were not exposed enough times for this to occur. Nor did we observe an increase in startle behaviour in any of our videos [26]. Performing a similar experiment and comparing the stress levels of individuals would help clarify to what extent any observed decisions may be stress related. Similarly examining individuals’ behaviours under noise treatment whilst in groups would also help separate stress or annoyance from the effects of masking noise. It is uncertain to what extent the reduction in foraging observed here and in other studies also occurs in the wild [27, 33], though this might be one explanation for the observed declines of populations in noisy areas [7, 38, 58].

Wild animals might also be negatively affected if masking noise increases the difficulty of detecting approaching predators [21, 22], although noise pollution might also have an adverse effect on predators by making it difficult for them to use auditory cues to detect prey [62]. In our study, birds did not seem to exhibit any preference for spending time in quiet, “safe” patches over noisy, “risky” patches. Although animals foraging in noisy areas have been shown to exhibit a greater tendency to retreat to cover or engage in other anti-predator behaviour when startled under noisier conditions [22, 24, 26], we did not observe this behaviour during our trials. This may lead to the suggestion that the changes in behaviour seen here are more due to the masking of conspecific acoustic signals than an increase in perceived risk of predation or stress. If this is the case, social species may be more severely impacted by masking noise than those that typically forage alone, though individuals could potentially avoid these impacts by altering their signals amplitude and pitch in response to adverse noise conditions [11–16]. Disentangling perceived predation risk from lack of access to auditory information from conspecifics will require further study, such as performing similar experiments on groups of animals.

Our experiment joins a growing number of studies showing how acoustic interference can influence on the behaviour of animals [2, 21, 32, 63]. Understanding how the disruption of auditory information by ambient noise affects behaviour will provide valuable insights into the utilisation of auditory information in animals and its importance in animal groups. Further study to distinguish between behaviour changing due to lack of auditory information or due to perceived predation risk will also determine if masking noise will have a greater impact on social species. All this could be extremely important when considering future management of species whose ranges are being encroached on by anthropogenic noise, such as those near shipping lanes or roads.

## Supporting information

S1 Fig Spectrogram of a section of the noise used in experiments [64]. (TIFF)

S1 File Time budget data. Time spent in different activities in different treatment types, for each trial.
(TXT)

S2 File Patch hoice data. Individual’s initial choice of test chamber for each trial.
(TXT)

S1 Table Model selection table for proportion of time spent in a treatments, in relation to treatment type, bird age and trial number. Full model and all models within Δ2 AICc of the top model are displayed. Most parsimonious model is highlighted in bold.
(PDF)

S2 Table Model selection table for probability of bird first entering a quiet chamber, in relation to bird age and trial number. Full model and all models within Δ2 AICc of the top model are displayed. Most parsimonious model is highlighted in bold.
(PDF)

S3 Table Model selection table for proportion of time spent foraging in treatments, in relation to treatment type, bird age and trial number. Full model and all models within Δ2 AICc of the top model are displayed. Most parsimonious model is highlighted in bold.
(PDF)

S4 Table Model selection table for proportion of time spent vigilant in treatments, in relation to treatment type, bird age and trial number. Full model and all models within Δ2 AICc of the top model are displayed. Most parsimonious model is highlighted in bold.
(PDF)
