# Supplementary figures and images for: Effects of ambient noise on zebra finch vigilance and foraging efficiency
Source: PLoS One. 2018 Dec 31;13(12):e0209471. doi: 10.1371/journal.pone.0209471 (PMC6312262; doi:10.1371/journal.pone.0209471)

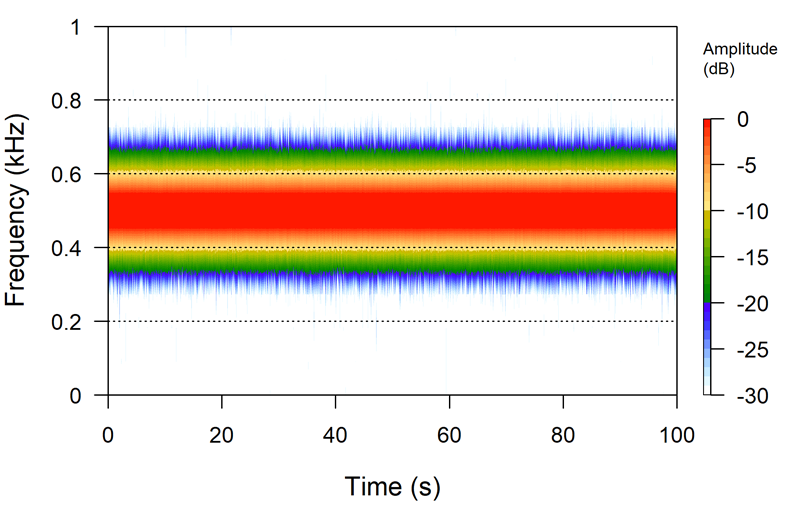

Supplement: S1 Fig — (TIFF) [file pone.0209471.s001.tiff]
