# Supplementary material for: Effects of ambient noise on zebra finch vigilance and foraging efficiency
Source: PLoS One. 2018 Dec 31;13(12):e0209471. doi: 10.1371/journal.pone.0209471 (PMC6312262; doi:10.1371/journal.pone.0209471)
Supplement: S1 Table — Full model and all models within Δ2 AICc of the top model are displayed. Most parsimonious model is highlighted in bold. (PDF) [file pone.0209471.s004.pdf]

Model ID	Candidate models	AICc	ΔAICc	df	Weight
Full	Snd+Tr+Age+Snd:Tr+Snd:Age+Tr:Age	43.2	32.47	9	
**1 (Null)**		**10.7**	**0**	**3**	**1**

Snd: Treatment type, *Tr:* Trial number, *Age*
