# Supplementary material for: Effects of ambient noise on zebra finch vigilance and foraging efficiency
Source: PLoS One. 2018 Dec 31;13(12):e0209471. doi: 10.1371/journal.pone.0209471 (PMC6312262; doi:10.1371/journal.pone.0209471)
Supplement: S2 Table — Full model and all models within Δ2 AICc of the top model are displayed. Most parsimonious model is highlighted in bold. (PDF) [file pone.0209471.s005.pdf]

Model ID	Candidate models	AICc	ΔAICc	df	Weight
Full	Tr+Age+Tr:Age	50.8	2.66	5	0
**1**	**Tr+Age**	48.1	**0**	**4**	**0.533**
2	Tr	48.4	0.26	3	0.467

Tr: Trial number, *Age*
