# Supplementary material for: Effects of ambient noise on zebra finch vigilance and foraging efficiency
Source: PLoS One. 2018 Dec 31;13(12):e0209471. doi: 10.1371/journal.pone.0209471 (PMC6312262; doi:10.1371/journal.pone.0209471)
Supplement: S3 Table — Full model and all models within Δ2 AICc of the top model are displayed. Most parsimonious model is highlighted in bold. (PDF) [file pone.0209471.s006.pdf]

Model ID	Candidate models	AICc	ΔAICc	df	Weight
Full	Snd+Tr+Age+Snd:Tr+Snd:Age+Tr:Age	-1.2	18.27	9	
**1**	**Snd**	-19.5	**0**	**4**	**0.692**
2	Snd+Tr	-17.8	1.62	5	0.308

Snd: Treatment type, *Tr :*Trial number, *Age*
